# Post-activation effects of accommodating resistance and different rest intervals on vertical jump performance in strength trained males

**DOI:** 10.1186/s13102-023-00670-y

**Published:** 2023-04-24

**Authors:** Sebastian Masel, Marcin Maciejczyk

**Affiliations:** Department of Physiology and Biochemistry, Faculty of Physical Education and Sport, University of Physical Education in Kraków, Kraków, Poland

**Keywords:** Variable resistance, Strength training, Post-activation performance enhancement, Trap bar deadlift, Power

## Abstract

**Background:**

Post-activation potentiation performance (PAPE) is a physiological phenomenon that has been studied numerously but the researchers are still seeking for the optimal application methods. The accommodating resistance was found to be an effective training method to acutely enhance subsequent explosive performance. The purpose of this study was to evaluate the effects of performing a trap bar deadlift with accommodating resistance on squat jump (SJ) performance with different rest intervals (90, 120, 150s).

**Methods:**

The study had a cross-over design and fifteen strength-trained males (age 22.9 ± 2.1 years; body height 182 ± 6.5 cm; body mass: 80.4 ± 9.8 kg; body fat 15.8 ± 7.0%; BMI 24.1 ± 2.8; lean body mass 67.5 ± 8.8 kg) participated in one familiarization, three experimental and three control sessions within three weeks. The conditioning activity (CA) used in the study was a single set of 3 repetitions of a trap bar deadlift at 80% 1RM with approximately 15% 1RM of an elastic band. The SJ measurements were performed at the baseline and post-CA after 90 or 120 or 150s.

**Results:**

The 90s experimental protocol significantly improved (p < 0.05, effect size 0.34) acute SJ performance whereas 120 and 150 s experimental protocols did not significantly improve performance. The following tendency was observed - the longer the rest interval, the smaller the potentiation effect; p value for 90s (0.046), 120s (0.166), 150s (0.745).

**Conclusions:**

A trap bar deadlift with accommodating resistance and 90s rest interval can be used to acutely enhance jump performance. A 90s rest interval was found to be optimal to enhance subsequent SJ performance, but the potential rest interval extension to 120s could also be taken by strength and conditioning coaches as the PAPE effect is highly individual. However, exceeding the rest interval to more than 120s may not be effective in optimising the PAPE effect.

## Introduction

Strength and conditioning coaches are constantly seeking optimal training methods to enhance power performance and one of them is using post-activation performance enhancement (PAPE) effect - a specific conditioning activity (CA) is applied prior to a similar movement task to obtain increased acute power. The enhancement is associated with potential mechanisms such as increased muscle temperature, muscle fiber water content and muscle activation [1] and is usually observed after 6–10 min after CA [1, 2] or 3–7 min considering specifically vertical jump performance [3]. However, the PAPE effect is highly individual and many factors need to be considered [2, 4, 5] to make it effective regarding training intervention (e.g. volume of a CA [6]) and characteristics of the individual (e.g. strength level [7]).

A variety of PAPE application methods were found to enhance performance. Any type of muscle contraction can be effective (only eccentric [8–10], isometric [10–14], eccentric-concentric [15] and only concentric [16]) as well as using additional training equipment such as flywheel devices [17, 18] or accommodating resistance [18–25]. It is important to determine the most efficient one for the individual as it should be as specific as possible to its sport. It could vary from e.g. an athlete warming up for a swimming competition having all possible training equipment (e.g. using a flywheel) or no equipment at all (e.g. using isometrics) to an athlete executing a strength and conditioning session in the gym to improve his power performance (e.g. using accommodating resistance). Introducing training intervention with a prolonged rest interval as suggested in the studies [1–3] between CA and a subsequent explosive task could diminish any potential benefits of PAPE as it could be too time-consuming and also influence training motivation. It was proved that an individual could effectively implement active recovery during an extended rest interval without losing the potentiation effect [26] but using accommodating resistance is also a well-described method that may allow reduction of the rest interval between CA and an explosive exercise [27]. Because time management is one of the crucial components of the training process, reducing the length of the rest interval to less than 3 min (suggested by Dobbs et al. [3] to be a minimum value for enhancing jump performance) may be especially important for strength and conditioning coaches. Therefore, the current evidence [19, 20, 22, 25] suggests that designing PAPE protocols with the use of accommodating resistance seems to be an optimal method in obtaining the potentiation effect with the simultaneous time management benefit.

The accommodating resistance method was repeatedly found to be effective in inducing PAPE [18–25]. With its ability to achieve greater velocity in the concentric portion of the lift and greater power output than using traditional resistance [28], it may allow enhanced performance with a relatively shorter rest interval of 90-120s between CA and a subsequent explosive task [19, 20, 22, 25]. Moreover, certain studies proved that the accommodating resistance was more effective in comparison to free weight resistance [21, 23, 25] in inducing PAPE. Even though a trap bar deadlift was suggested as being an effective training alternative to a squat, [29] there is little evidence of a trap bar deadlift inducing PAPE [30–34]. The results of the studies using only traditional resistance are not consistent - two of them showed no PAPE effect [30, 31], whereas one of them enhanced subsequent explosive performance. Furthermore, a trap bar deadlift was more effective compared to a back squat [32]. Additionally, there are two studies [33, 34] where the accommodating resistance was used while performing a trap bar deadlift. Both of them [33, 34] involve a vertical jump component as an explosive exercise. It may be especially important as monitoring vertical jump height is a method for evaluating the effectiveness of the training program [35]. The first one [33] was not effective in enhancing subsequent countermovement jump (CMJ) performance and the second [34] was partially effective and showed a higher effect for a squat jump (SJ) than CMJ as a higher percentage of the players responded positively (improvement in absolute values by ≥ 0.8 cm between baseline and post-CA jumps) in SJ (73%) than CMJ (50%). Therefore, more studies are necessary to evaluate the real potential of a trap bar deadlift with accommodating resistance as a CA to reduce the rest interval between CA and a subsequent explosive task.

Even though a trap bar deadlift is a frequently used exercise, the current evidence of its use with accommodating resistance on PAPE is very limited and so far the outcome has been negative. Therefore, the main objective of this study was to evaluate the effects of performing a trap bar deadlift with accommodating resistance as a CA on jump performance with rest intervals shorter than 3 min between CA and a subsequent explosive task. An additional purpose of the study was to determine if a trap bar deadlift combined with accommodating resistance could be an effective CA as the current evidence did not support it [33, 34]. SJ was implemented in the study as it starts from an isometric position as well as a trap bar deadlift. It was hypothesized that PAPE could be induced with rest intervals shorter than 3 min.

## Materials and methods

### Study design

**Fig. 1:**
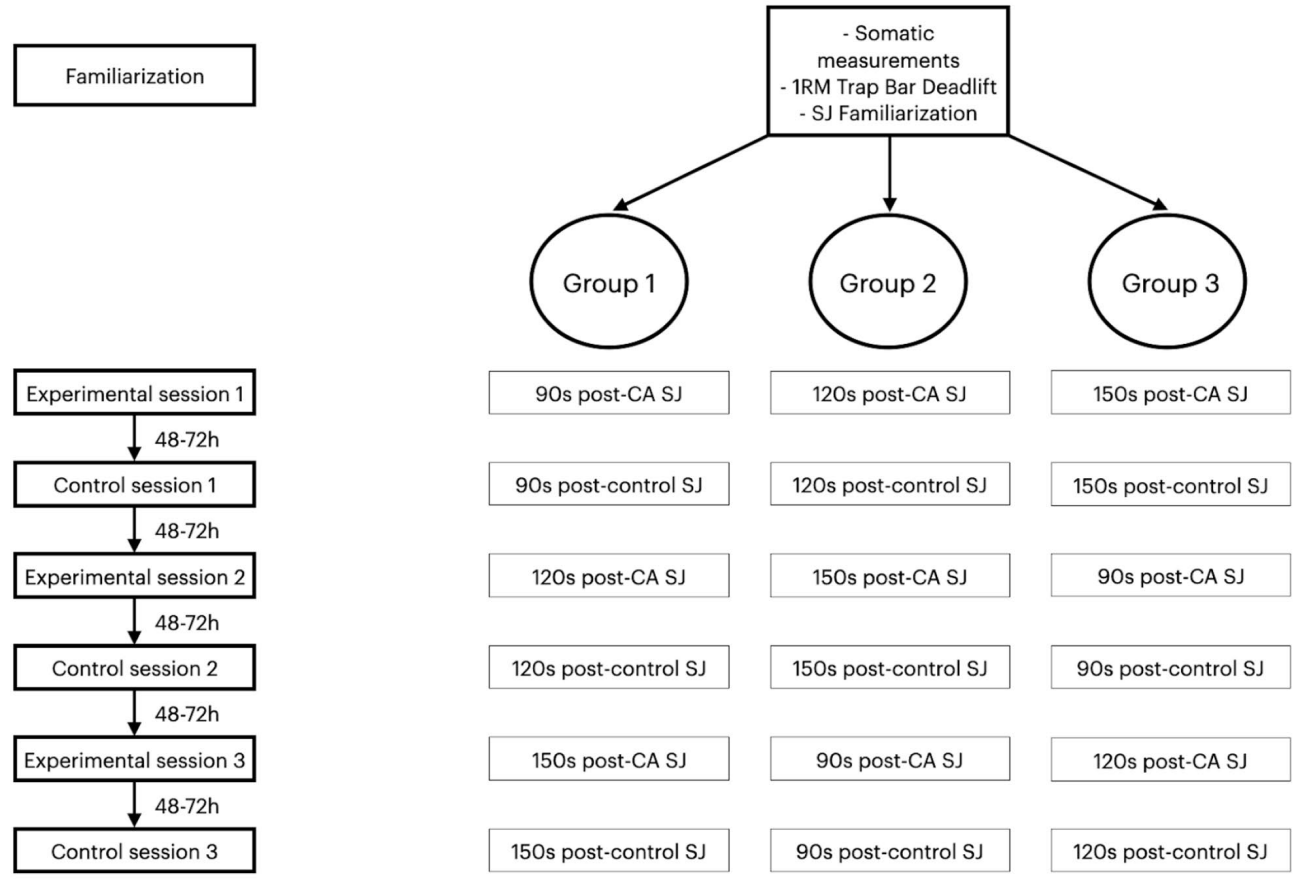
Study design. 1 RM - one repetition maximum; CA - conditioning activity; SJ - squat jump

It was a cross-over study and the participants took part in one familiarization, three experimental and three control sessions within three weeks. After the familiarization session, to introduce randomization, participants were divided into three groups of five participants and performed the study in three different orders (Fig. 1). Randomization was carried out in a following manner: during the familiarization session each of the participants chose one of three scraps of paper with unseen “G1”, “G2” or “G3” and was assigned to perform the study in that order. All daily sessions were performed at a similar time of day (from 8 a.m. to 12 a.m.) with 48-72 h apart and it was the first participants’ physical activity of the day. The first experimental session was performed after 72-96 h after a familiarization session due to intensity of the measurements. The familiarization session included somatic measurements, determination of one-repetition maximum (1RM) in a trap bar deadlift and familiarization with a squat jump (SJ) test. The experimental sessions included a standardized warm-up, baseline SJ, PAPE condition with CA and post-CA SJ (after 90 or 120 or 150s); the control sessions included a standardized warm-up, baseline SJ, control condition without CA and post-control SJ (after 90 or 120 or 150s). Conditioning activity used in the study was a single set of 3 repetitions of a trap bar deadlift at 80% 1RM with approximately 15%1RM of an elastic band and the rest of the load was provided by traditional resistance.

There were the following inclusion criteria: (a) regular participation in strength training (at least 3 times a week); (b) relative strength level in a trap bar deadlift ≥ 1.5 kg/body mass; c) lack of injuries or other health contraindications in the last 6 months. Participants were recruited in the following manner: an announcement of the recruitment of volunteers was carried out with the aims of the study and inclusion criteria and therefore, the participants eligible for the study were chosen to participate. Participants were instructed to follow their normal dietary, supplement, training and sleeping habits during the study. All participants were informed about the study protocol, benefits and potential risks of the study. They voluntarily took part in the experiment, providing signed informed consent and were allowed to withdraw from the experiment at any moment. The Bioethics Committee accepted the study protocol (Regional Medical Chamber in Kraków, opinion no: 1/KBL/OIL/2022) which was performed according to the ethical standards of the declaration of Helsinki 2013.The sample size was calculated a priori using G*Power 3.1 statistical software (Dusseldorf, Germany) with the following variables: the ANOVA with repeated measures, an effect size (f) of 0.5, an alpha value of 0.05, a statistical power of 0.95 (95%) and a correlation between measurements of 0.50. A minimum sample size of 15 individuals was obtained.

### Participants

Participants of the study were fifteen strength-trained males (age 22.9 ± 2.1 years; body height 182 ± 6.5 cm; body mass: 80.4 ± 9.8 kg; body fat 15.8 ± 7.0%; BMI 24.1 ± 2.8; lean body mass 67.5 ± 8.8 kg) with various sport backgrounds (6 in volleyball, 3 in football, 1 in powerlifting, 1 in fencing, 1 in sprinting, 1 in cycling, 1 in crossfit, 1 in calisthenics). Originally, sixteen participants were willing to participate in the study but one participant was excluded from taking part in the study after 1RM measurements due to an insufficient relative strength level (approximately 1.4 kg/body mass).

### Warm-up

Each session started with a standardized warm-up that included: 10 min of cycling on a cycle ergometer (Monark, Sweden) at a heart rate of 100–120 bpm; then a set of dynamic stretching was performed which consisted of 3 exercises of 10 repetitions each: knee to chest with calf raise; heel to buttocks with calf raise; hip external rotation with calf raise. Total duration of the standardized warm up was approximately 15 min.

### Familiarization session

The familiarization session began with the somatic measurements - body height was measured using a stadiometer (SECA, Germany) whereas body mass and body composition (body fat and lean body mass) were measured using the JAWON scale (Korea, bioelectrical impedance analysis). All the measurements were performed barefoot and participants were instructed to stand still and distribute their body weight evenly on the platform.

After somatic measurements, 1RM determination in a trap bar deadlift was executed as previously described [34]. Participants performed a standardized warm-up and one minute after the standardized warm-up participants began performing a trap bar deadlift warm-up, starting with 10 repetitions with a load of 25 kg. After that, participants performed 3 to 4 sets of 3 repetitions, increasing the load with each set by 10–15% until they reached approximately 80% of an estimated 1RM. Then participants performed solely 1 repetition with an increased load by 5–10 kg for each subsequent attempt until they reached their 1RM (were unable to perform a lift with a proper technique). Sets of 3 repetitions included rest intervals of three minutes, whereas rest intervals between single repetition sets were 4–5 min. The participants were instructed to perform each repetition with a maximal velocity in the concentric phase of the lift and controlled eccentric phase (approximately 2s of eccentric phase). All repetitions were performed from the floor level (with high handles of a trap bar). The mean relative 1RM in a trap bar deadlift amounted to 2.01 ± 0.27 kg/body mass.

After the 1RM determination, the participants performed the familiarization with the squat jump test. Each of the participants executed the SJ test 3 to 5 times depending on how quickly the participant learned the movement pattern.

### Squat jump measurement

Jumping tests were performed using OptoJump (Italy) technology - an optical measurement system that consists of a transmitting and receiving bar and was shown to be a valid and reliable tool for the assessment of vertical jump height [36]. SJ testing was performed as previously described [34]. During SJ, participants were instructed to perform a downward movement until they reach approximately 90° of knee flexion, then an isometric hold of 2 s and a jump from an isometric position. All the jumps were performed with arms placed on the hips and participants were forbidden to move them during the test. Because SJ is a test from an isometric position, participants were forbidden to perform another downward movement after an isometric hold of 2 s. The participants were allowed to choose the width of their stance while performing a test. During the familiarization with the test and throughout the whole duration of the study, the isometric hold at the bottom of the squat was counted and the jumping command was verbalized (“1… 2… JUMP”) by the supervisor of the study to avoid improper execution of the test (34).

### Experimental and control sessions

**Fig. 2:**
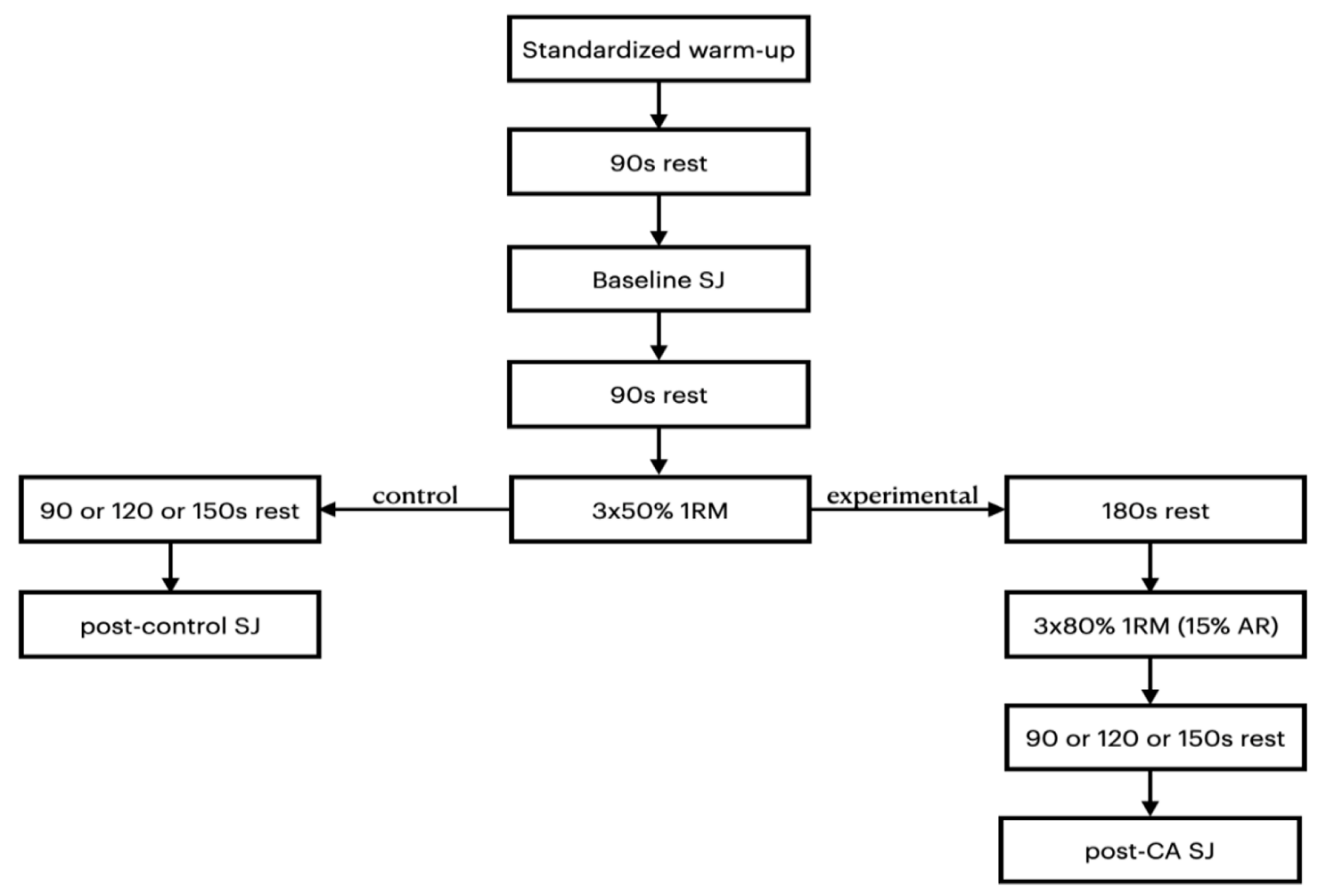
Study flow

After the familiarization session, the participants performed three experimental and three control sessions. Control sessions took approximately 25 min and experimental sessions approximately 30 min. The participants began each session with an identical standardized warm-up and 90s after the warm-up performed baseline SJ. Then, 90s after baseline SJ, they performed a single set of 3 repetitions at 50% 1RM. In control protocols, depending on the day, participants performed post-control SJ after 90 or 120 or 150 s. In experimental protocols, after 180 s of recovery, participants performed a conditioning activity of the study - a single set of 3 repetitions of a trap bar deadlift at 80% of 1RM with approximately 15% 1RM of an elastic band. Then, depending on the day, participants performed post-CA SJ after 90 or 120 or 150 s (Fig. 2).

To assess an adequate accommodating resistance, four types of brand new (to avoid potential loss of band tension) elastic bands of different tension were used throughout the study. The resistance of the band was calculated as the median of the range of the resistance suggested by the producer. The thickness of a band was appropriate to a participant performing a CA in addition to a traditional resistance in obtaining the intended percentage of 1RM. Throughout the protocols, two repetitions of SJ were performed in the same manner as described in the section before and the repetition with a higher value of jump height (JH) was kept for the statistical analysis.

### Statistical methods

All data is presented as mean and standard deviation (SD). Data distribution was checked using the Shapiro–Wilk test. Homogeneity of variance within the groups was tested via Levene’s test (variance of the analyzed parameters was similar in both groups). The ANOVA with repeated measures (analyzed factors: condition [PAPE vs. control], time [baseline vs. post] and interaction between these factors) was used to assess significance of the effect of CA on changes in jump performance. In the case of a significant influence of the main factor (ANOVA, p < 0.05), post hoc analysis was performed using the LSD test. The differences in all analyzed indices were considered statistically significant at the level of p < 0.05. The effect size (Cohen’s d) was calculated and interpreted as small (0.20), medium (0.50), or large (0.80) [37]. Statistical analysis was performed using Statistica 12.0 software (StatSoft, Tulsa, OK, USA).

## Results

Analyzing the data, a significant interaction was observed in all the parameters of the jump in PAPE condition with a 90s rest interval (p = 0.046). Conditions with 120s (p = 0.166) and 150s (p = 0.745) did not significantly improve JH. Post-hoc analysis indicated significant changes in baseline versus post measurements in the PAPE condition, whereas the control condition did not indicate it (Table 1).

However, it is worth noting that the results of PAPE showed the following tendency - the longer the rest interval, the smaller the potentiation effect. Also, pre to post-CA changes in mean values in JH are similar for 90s (1.5 cm; 36.6 ± 4.3 to 38.1 ± 4.4) and 120s PAPE conditions (1.2 cm; 36.4 ± 4.5 to 37.6 ± 4.4).

**Table 1:** Results of jumping tests after applicated CA with different (90, 120, 150s) rest intervals (presented as mean ± SD)

Variable	Condition	Baseline	Post	Effect: condition F(p)	Effect: Time F(p)	Interaction F(p)	p: post-hoc pre-post (Cohen’s d)
**90s**							
JH (cm)	PAPE	36.6 ± 4.3	38.1 ± 4.4	0.174 (0.680)	3.030 (0.092)	4.342 (0.046)	0.01 (0.34)
	CNTR	36.7 ± 4.7	36.6 ± 5.0				0.81 (0.02)
FT (s)	PAPE	0.545 ± 0.032	0.557 ± 0.033	0.204 (0.655)	2.484 (0.126)	4.371 (0.046)	0.01 (0.37)
	CNTR	0.546 ± 0.036	0.544 ± 0.038				0.72 (0.05)
RAP (W/kg)	PAPE	15.2 ± 1.0	15.8 ± 1.0	0.429 (0.517)	4.406 (0.044)	4.212 (0.049)	0.01 (0.6)
	CNTR	15.3 ± 1.0	15.3 ± 1.3				0.97 (0)
**120s**							
JH (cm)	PAPE	36.4 ± 4.5	37.6 ± 4.4	0.076 (0.785)	3.286 (0.079)	2.022 (0.166)	NS
	CNTR	36.5 ± 4.6	36.6 ± 4.6				NS
FT (s)	PAPE	0.544 ± 0.033	0.553 ± 0.033	0.090 (0.766)	2.884 (0.100)	2.095 (0.158)	NS
	CNTR	0.545 ± 0.034	0.545 ± 0.0.34				NS
RAP (W/kg)	PAPE	15.2 ± 1.3	15.6 ± 1.3	0.111 (0.741)	3.582 (0.068)	1.312 (0.261)	NS
	CNTR	15.2 ± 1.3	15.3 ± 1.4				NS
**150s**							
JH (cm)	PAPE	36.7 ± 5.1	36.8 ± 4.6	0.021 (0.885)	0.489 (0.490)	0.107 (0.745)	NS
	CNTR	36.3 ± 5.0	36.7 ± 5.1				NS
FT (s)	PAPE	0.546 ± 0.038	0.547 ± 0.035	0.028 (0.867)	0.453 (0.506)	0.042 (0.839)	NS
	CNTR	0.543 ± 0.037	0.545 ± 0.038				NS
RAP (W/kg)	PAPE	15.4 ± 1.3	15.5 ± 1.2	0.086 (0.771)	0.831 (0.369)	0.153 (0.699)	NS
	CNTR	15.3 ± 1.4	15.3 ± 1.4				NS

JH - jump height; FT - flight time; RAP - relative average power; NS (non-significant).

## Discussion

The results of our study showed that the rest interval of 90s was effective in enhancing subsequent jump performance but the extension of the rest interval to 120-150s was not effective. A single set of a trap bar deadlift with accommodating resistance as a CA can be an effective way to enhance subsequent explosive performance with a relatively short rest interval (90s) between these activities.

To our knowledge, this is the first study that examined the use of accommodating resistance and various rest intervals shorter than 3 min (90, 120, 150s) between CA and a subsequent explosive exercise. Additionally, a trap bar deadlift was used as a CA that at this point was not excessively studied regarding PAPE. Our results are in agreement with multiple studies proving a positive influence of the use of accommodating resistance in enhancing subsequent explosive performance [18–25]. Originally, the meta-analysis by Wilson et al. [2] suggests using rest intervals of 6–10 min for the PAPE effect to occur and the meta-analysis by Dobbs et al. [3] 3–7 min, considering subsequent vertical jump performance. However, this study confirms the other data [19, 20, 22, 25] where the use of accommodating resistance allowed reduction of the rest interval between the CA and the subsequent explosive task to less than 180 s. One of the potential explanations of this phenomenon may be that the use of accommodating resistance generated the lesser fatigue than with traditional resistance and therefore allowed the potentiation effect to occur faster.

Our study also proved that extending the rest interval, between a CA and subsequent vertical jump to more than 90s had the following tendency - the longer the rest interval, the smaller the potentiation effect. A significant difference was detected between the baseline and post-CA jumps for 90s condition, close to a significant difference for 120s condition and far from being significant for 150s condition. The use of accommodating resistance and the rest interval of 90s was already proved to be effective using a squat as a CA [19, 22] and not effective using a trap bar deadlift as a CA [33, 34]. The rest interval of 120s was effective once [21] and not effective once [25], whereas the rest interval of 150s was not studied at this point. Even though the performance improvements using a rest interval of 120s were not statistically significant, it can be observed that there is a small difference between pre to post-CA changes in mean values for 120s and 90s (1.5 to 1.2 cm).

An interesting observation is that the exact rest interval (90s) with the same CA proposed in the other study [34] that was not effective in inducing PAPE turned out to be the only one significantly improving performance in this study. The authors suggested that a potential limitation was a sample size, as only 11 participants performed a condition with SJ. However, in this study the required sample size was calculated and the number of 15 individuals was obtained. In the above-mentioned study [34] it was impossible to calculate the required sample size as the participants needed to perform the same type of a training program to meet the criteria of the homogeneity of the group. The other difference between these studies is the use of four types of elastic bands in this study in contrast to only one used in the previous one [34]. That could allow adjusting more effectively a load to a given individual that is a key element in optimizing PAPE as the intensity of a CA is an important component of an effective PAPE protocol [2]. Additional considerations were made regarding the results of the second study that examined the use of a trap bar deadlift and accommodating resistance [33]. That study [33] used very high intensity of a CA (70% of free-weight resistance and 23% of accommodating resistance) that could generate excessive fatigue with a combination of short rest intervals (30, 90, 180s) in subsequent CMJ. Also, one could speculate if the group was sufficiently homogenous as there is a wide discrepancy considering 1RM measurements. The relative strength level in a trap bar deadlift was presented as 1.78 ± 0.41 meaning there could be individuals not having sufficient relative strength level suggested by Seitz and Haff [7] to enhance PAPE. It was suggested that stronger individuals are able to express the PAPE effect earlier than weaker individuals [27]. That is exceptionally important considering the use of accommodating resistance and potential reduction of the rest interval between CA and a subsequent explosive task. Thus, the improved methodology of this study seems to be a critical reason for achieving a positive outcome with this type of CA.

As the use of accommodating resistance in PAPE was confirmed in the previous studies [19, 20, 22, 25] in reducing the rest interval in comparison with original recommendations [1–3], an idea to reduce the length of the rest interval to less than 90s could be an interesting direction for future research. Previous research by Wyland et al. [21] reported that a 60s rest interval with a CA of 5 sets of 3 repetitions of a back squat at 85% of 1RM (with 30% of the total resistance coming from elastic bands) did not enhance subsequent sprinting performance. On the contrary, the study by Mina et al. [23] where the same type of a CA was used as in the study by Wyland et al. [21] allowed to enhance subsequent CMJ after only 30s. The study by Scott et al. [33] also used the 30s rest interval but there was no PAPE effect. Possibly, the ideal combination for strength and conditioning practitioners would be to limit the rest interval to a minimum, optimally performing a subsequent explosive task right after a CA with the potentiation effect. Two studies [21, 25] investigated an immediate response (within 15 s) after a CA and both of them failed to show PAPE effect after such a short rest interval.

Accommodating resistance was proved to acutely enhance subsequent explosive performance in less than 180 s [19, 20, 22, 25] but there is no evidence which mechanisms allow shortening of the rest interval. Tillin and Bishop [4] stated that to determine potential PAPE response, an appropriate balance is necessary between type and parameters of the CA and fatigue induced by the CA. Excessive fatigue induced by the CA seems to be detrimental for subsequent explosive performance. Training status, load, mode and sets all potentially influence the PAPE response, but the length of the rest interval may be the most important component of the PAPE protocols [3]. Thus, the rest interval needs to be applied appropriately depending on the type of a CA. Wallace and Bergstrom [38] proposed potential mechanisms of accommodating resistance efficacy and one of them is reducing the large deceleration period of the concentric phase. It could explain why the use of accommodating resistance in the CA seems to generate less fatigue and allows us to observe the potentiation response in less than 180 s. In this study we did not evaluate the possible mechanisms of the observed phenomenon and that could be the subject of future studies.

This study has a practical recommendation for the practitioners that an enhancement effect is likely to occur in the 90-120s window after this type of CA and the additional extension of the rest interval seems to be sub-optimal. PAPE has an individual response and in fact various loading strategies may be effective in enhancing performance This is an important recommendation as it may allow avoidance of testing different protocols on the athletes before implementing this type of training method into their training program. However, despite the PAPE effect occurring in this study, the results should be applied with caution as the participants were not professional athletes. Their relative strength level (2.01 ± 0.27 kg/body mass) matches the recommendations made by Seitz et al. [7] but not an ideal homogeneity of the group (different sport background) could be a potential limitation of the study. Additionally, in any further investigation researchers should consider determining the exact band tension in addition to having brand new elastic bands to match the intended training intensity as accurately as possible.

## Limitations of the study

The study protocol did not involve the exact determination of the band tension. Even though the bands used in the study were new, the band tension could slightly vary between the participants due to different anthropometrics (body height). Additionally, in future studies researchers should try to recruit the participants within the same sport. A further investigation is needed to examine if the use of accommodating resistance could also be effective with rest intervals shorter than 90s. This type of research project would probably require testing different loading interventions such as various volumes and intensities of a CA and different percentages of 1RM coming from elastic bands.

## Conclusions

A single set of a trap bar deadlift (three repetitions at 80% 1RM) with the use of accommodating resistance and 90s rest interval was effective in enhancing SJ performance in strength-trained males. Additionally, the following tendency could be observed - the longer the rest interval, the smaller the potentiation effect; p value for 90s (0.046), 120s (0.166), 150s (0.745). Thus, strength and conditioning specialists should consider not exceeding 120s rest interval with this type of CA in order to optimise the PAPE effect.

## Data Availability

The datasets analyzed during the study are available from the corresponding author (SM) on reasonable request.

## List of abbreviations

PAPE: Post-activation potentiation enhancement
CA: Conditioning Activity
1RM: One repetition maximum
CMJ: Counter-movement jump
SJ: Squat jump
